# Identification of Chromosomal Errors in Human Preimplantation Embryos with Oligonucleotide DNA Microarray

**DOI:** 10.1371/journal.pone.0061838

**Published:** 2013-04-16

**Authors:** Lifeng Liang, Cassie T. Wang, Xiaofang Sun, Lian Liu, Man Li, Craig Witz, Daniel Williams, Jason Griffith, Josh Skorupski, Ghassan Haddad, Jimmy Gill, Wei-Hua Wang

**Affiliations:** 1 Houston Fertility Institute, Houston, Texas, United States of America; 2 Key Laboratory of Major Obstetrics Diseases of Guangdong Province, The Third Hospital Affiliated to Guangzhou Medical University, Guangdong, China; 3 Pacgenomics Inc., Village Medical Center, Thousand Oaks, California, United States of America; State Key Laboratory of Reproductive Biology, Institute of Zoology, Chinese Academy of Sciences, China

## Abstract

A previous study comparing the performance of different platforms for DNA microarray found that the oligonucleotide (oligo) microarray platform containing 385K isothermal probes had the best performance when evaluating dosage sensitivity, precision, specificity, sensitivity and copy number variations border definition. Although oligo microarray platform has been used in some research fields and clinics, it has not been used for aneuploidy screening in human embryos. The present study was designed to use this new microarray platform for preimplantation genetic screening in the human. A total of 383 blastocysts from 72 infertility patients with either advanced maternal age or with previous miscarriage were analyzed after biopsy and microarray. Euploid blastocysts were transferred to patients and clinical pregnancy and implantation rates were measured. Chromosomes in some aneuploid blastocysts were further analyzed by fluorescence in-situ hybridization (FISH) to evaluate accuracy of the results. We found that most (58.1%) of the blastocysts had chromosomal abnormalities that included single or multiple gains and/or losses of chromosome(s), partial chromosome deletions and/or duplications in both euploid and aneuploid embryos. Transfer of normal euploid blastocysts in 34 cycles resulted in 58.8% clinical pregnancy and 54.4% implantation rates. Examination of abnormal blastocysts by FISH showed that all embryos had matching results comparing microarray and FISH analysis. The present study indicates that oligo microarray conducted with a higher resolution and a greater number of probes is able to detect not only aneuploidy, but also minor chromosomal abnormalities, such as partial chromosome deletion and/or duplication in human embryos. Preimplantation genetic screening of the aneuploidy by DNA microarray is an advanced technology used to select embryos for transfer and improved embryo implantation can be obtained after transfer of the screened normal embryos.

## Introduction

Aneuploidy is one of the most crucial factors affecting embryo implantation and is also a major cause of birth defects [1], [2]. It has been reported that the aneuploidy rate is extremely high in patients with repeated implantation failure [3], recurrent miscarriages [4], previous aneuploid conceptions [5] and advanced maternal age [2], [6]–[9]. Recently it has been found that a high aneuploidy rate (∼40%) is also present in younger (∼31 yrs old) patients undergoing in vitro fertilization (IVF) [10], [11]. Preimplantation genetic screening (PGS) by 24-chromosome microarray is an important diagnostic method to identify aneuploidy and other chromosome abnormalities [12]. Transfer of euploid blastocysts has significantly increased clinical pregnancy and embryo implantation rates [11], [13], [14].

Currently, two major microarray platforms are used for PGS in human IVF. One is a bacterial artificial chromosomes (BAC) microarray platform provided by BlueGnome [11], [14]–[16] and the other is a single-nucleotide polymorphisms (SNP) microarray platform provided by Affymetrix and Illumina [17]–[19]. It has been reported that the BAC platform uses a tiling-resolution microarray encompassing 32K overlapping BAC clones selected to cover the entire human genome, while the Affymetrix 100K SNP array contains 35-mer oligonucleotides with a total of 116, 204 SNPs [12], [20]–[23]. Both platforms have been successfully used in human PGS in which only one cell or few cells are biopsied from an embryo [11], [13], [14], [19], [24].

Previous studies comparing the performance of different platforms for DNA array found that the oligo microarray platform provided by NimbleGen containing 385K isothermal probes had the best performance when evaluating dosage sensitivity, precision, specificity, sensitivity and copy number variations (CNVs) border definition [20]–[23]. The most up-to-date NimbleGen array platform has 4.2 million probes per array that is able to detect CNVs down to ∼5 kb, and it is the most sensitive DNA microarray platform developed. Therefore, identification of very small gains and losses in chromosomes from single cells is possible [21], [22], [25], [26]. Previously we had limited data to show that NimbleGen array platform was able to provide similar chromosome information for aneuploidy screening in human embryos when compared with the BAC array platform [14]. Although the NimbleGen microarray platform has been used in some research fields and clinics [21], [22], [25]–[28], it has not been used for aneuploidy screening in human preimplantation embryos. In the present study, we used the NimbleGen oligo microarray platform to perform aneuploidy screening in human blastocysts obtained from patients undergoing IVF and PGS. The study aimed to evaluate the application of a new array platform with more probes to cover more genomic regions in human PGS. Using this new array platform, it may be possible to identify minor chromosomal abnormalities.

## Materials and Methods

### Ethics statement

Patients undergoing IVF and PGS signed written consents for embryo biopsy and aneuploidy screening. When the patients signed the consents, they were aware that embryo biopsy and PGS are investigational procedures requiring removal of one or more cells from embryos and that the genetic analysis of their samples would be used for selection of euploid embryos for transfer and for investigational purposes. As promulgated by the United States Department of Health and Human Services, this study was exempted from Institutional Review Board approval as it involved the review of existing data, documents, records, and diagnostic specimens in such a manner that subjects could not be identified directly or through identifiers linked to the subjects.

### Patient preparations for egg retrieval and PGS

Patients underwent PGS because they had either previously experienced recurrent miscarriage and/or were of advanced maternal ages. For controlled ovarian hyperstimulation, patients were treated with a mixed protocol of human menopausal gonadotropin and a GnRH antagonist. The follicle stimulation hormone products (Follistim, Gonal-F, or Bravelle plus Menopure) were usually started within the first 2–3 days after the period begins with a starting dose between 150 and 375 iu per day. The dose was adjusted as the stimulation progressed. Human chorionic gonadotropin (hCG), at a dose of 250 mcg, was injected to induce final oocyte maturation when at least two dominant follicles reached a diameter of >18 mm. Eggs were retrieved via transvaginal ultrasound between 35–37 hrs after hCG administration.

### Embryo culture and embryo biopsy

**Figure 1:**
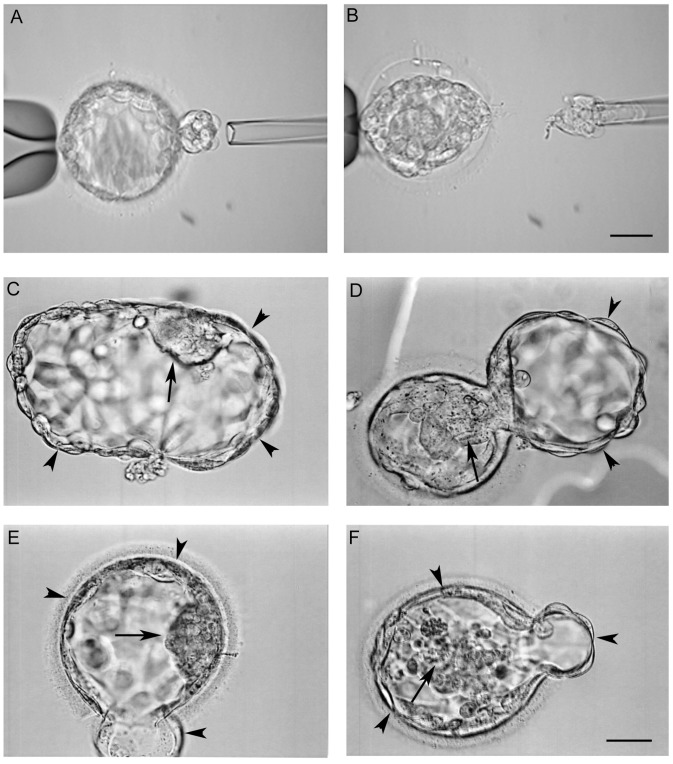
Microscopic images of human blastocysts for biopsy and recovering after vitrification/warming. (A) Some trophectoderm cells in a blastocyst started to hatch and (B) the trophectoderm cells were biopsied with assisted laser cutting. Images in C–D show the blastocysts after vitrification and warming. Blastocysts had been cultured for 2–4 hrs after warming, showing good (ICM and trophectoderm cells) hatched (C) and hatching (D) blastocysts. The hatching blastocyst in (E) has good ICM but fair trophectoderm while the blastocyst in (F) has both fair ICM and trophectoderm. Arrows indicate ICMs and arrow heads indicate trophectoderm cells. Bar = 40 µm.

Oocytes were cultured in Global^™^ medium (IVFonline, CT, USA) supplemented with 10% serum protein substitute (SPS, IVFonline) for 4–5 hrs before removing the surrounding cumulus cells in a HEPES buffered medium (Global-HEPES) containing 40 iu hyaluronidase. The mature (metaphase II) oocytes were inseminated by intracytoplasmic sperm injection (ICSI). Fertilization was examined 16–18 hrs after ICSI and zygotes were cultured in Global medium supplemented with 10% SPS at 37°C in a humidified atmosphere of 5.5% CO_2_, 5% O_2_ and balanced nitrogen until day 6 after insemination. At Day 3, a hole about 20 µm was opened in the zona pellucida using the ZILOS-tk^™^ laser system (Hamilton Thorn Bioscience Inc., MA, USA). On Day 5, embryos were examined with an inverted microscope and if trophectoderm (TE) cells started to hatch from the opening in the zona pellucida (Figure 1A), some hatched TE cells (∼10) were biopsied using a 20 µm polished biopsy pipette with assisted cutting by the laser (Figure 1B). Blastocyst biopsy was performed on TE cells at days 5 and 6 depending on blastocyst development. After biopsy, the embryo proper was cultured in Global medium supplemented with 10% SPS for 1–2 hrs before vitrification. The biopsied cells were washed with a washing buffer, placed in tubes with cell lysis buffer and were then frozen at –20°C before being processed for microarray.

### Blastocyst vitrification

Blastocysts were vitrified after the blastocoele completely collapsed according to a previous method reported by Mukaida *et al*. [29] by using Irvine vitrification kit (Irvine Scientific, Irvine, CA USA). Briefly, blastocysts were equilibrated in the equilibration solution contained 7.5% (v/v) ethylene glycol and 7.5% (v/v) dimethylsulphoxide for 2 minutes on a warming stage (37°C). The blastocysts were then transferred into the vitrification solution that was composed of 15% (v/v) ethylene glycol, 15% (v/v) dimethylsulphoxide and 0.5 M of sucrose and then loaded onto a vitrification straw within 45 seconds. The straw was immediately plunged into the protective straw inside liquid nitrogen for cryopreservation. All embryos were vitrified individually and then stored in the liquid nitrogen until warming for frozen embryo transfer (FET).

### Microarray with oligo NimbleGen platform

Biopsied TE cells were lysed and the cell’s genomic DNA was amplified using Rubicon whole-genome amplification kit (Rubicon, MI, USA). Amplified samples were purified with a GenElute PCR Clean-Up Kit (Sigma, MO, USA). The DNA concentration of purified samples was measured using Nanodrop 2000 (Thermo, DE, USA) and the samples were then labeled with Cy3 using the NG dual color labeling kit according to manufacturer’s instructions (Roche NimbleGen, IN, USA). Labeled samples were mixed with Cy5 control labeled samples, dried, dissolved and loaded on NimbleGen 6×630K comparative genome hybridization (CGH) tiling array following the NimbleGen hybridization protocol. After overnight hybridization, arrays were washed following the NimbleGen washing protocol. Arrays were dried and scanned with a NimbleGen MS200 scanner (Roche NimbleGen, IN, USA) at 2 µM scanning resolution. Scanned images were analyzed by Deva 1.1 software (Roche NimbleGen, IN, USA) and the normalized ratio of each sample versus the control was retrieved following the NimbleGen CGH data analysis protocol. Finally the normalized ratio of each sample was input into Nexus 6.1 software (Biodiscovery, CA, USA) and the Log2 ratio result of each sample’s whole genome view is presented.

### Blastocyst warming and FET

For warming, normal euploid blastocysts were exposed to 1 M warmed (37°C) sucrose for 1 minute. Blastocysts were then transferred to 0.5 M sucrose for 3 minutes and to a basic solution (HEPES buffered tissue culture medium 199 supplemented with 20% SPS) for 10 minutes with a solution change after 5 minutes at room temperature. After completion of the warming process, blastocysts were washed with Global medium supplemented with 10% SPS and then cultured in the same medium for 2–4 hrs before transfer. Blastocyst quality was assessed using standard assessments developed by the Society of Assisted Reproductive Technology [30]. As shown in Figure 1C–F, two different categories of inner cells mass (ICM) and TE cells were used: ICM: good (many cells, tightly compact and distinct ICM) and fair (several cells, loosely grouped); TE: good (many cells, forming a cohesive layer) and fair (few cells, forming a loose epithelium).

Patients for FET received oral micronized estradiol and transdermal estrogen for preparation of the endometrium for 12–20 days. Intramuscular administration of progesterone in oil was then initiated 6–7 days before embryo transfer and was continued until the first pregnancy test two weeks after embryo transfer. Ongoing pregnancies were supported by continued estradiol and progesterone.

### Pregnancy diagnosis

Fourteen days after embryo transfer, pregnancy was checked by a serum β-hCG assay. When the β-hCG was >5 mIU/mL the patients were regarded as having a biochemical pregnancy. Four weeks after embryo transfer, when a gestational sac and a heart beat appeared ultrasonographically, the patients were diagnosed as having a clinical pregnancy.

### Assessment of abnormal chromosomes in the aneuploid blastocysts with fluorescence in-situ hybridization (FISH)

Ten aneuploid blastocysts detected by microarray were fixed on glass slides and then processed for FISH. Because the errors in the chromosomes were known, the probes for FISH were chosen based on the abnormal chromosomes and sex chromosomes only. Fluorescence signals for FISH were examined under a fluorescence microscope. The data obtained by FISH and microarray was further compared to evaluate the accuracy of these techniques.

### Statistical analysis

Interval data was analyzed by one-way analysis of variance and categorical differences between groups were analyzed by Chi-square. If the P value was less 0.05, it was considered to be statistically different.

## Results

### NimbleGen oligo array can detect minor chromosomal abnormalities

**Figure 2:**
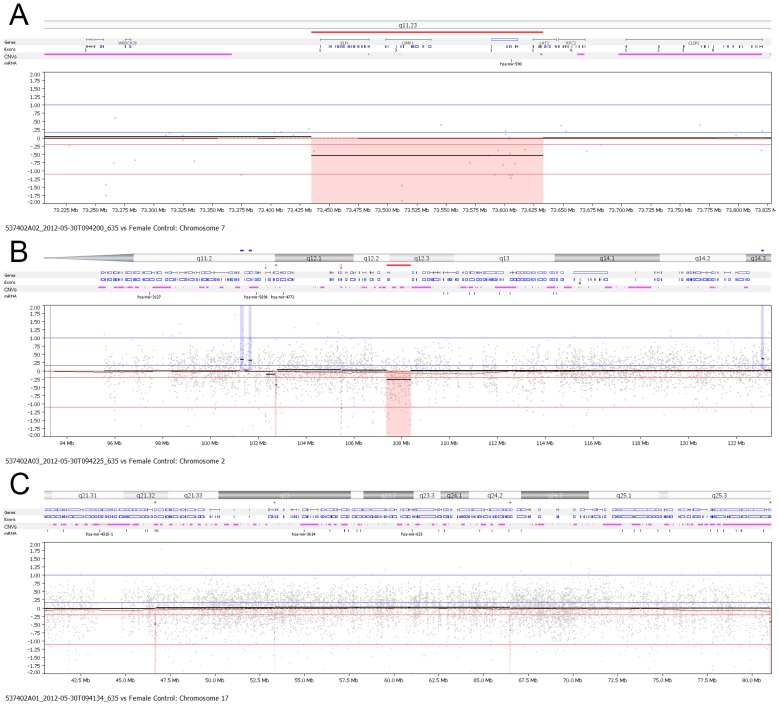
Sensitivity detection by NimbleGen oligo microarray. Three samples with known length of chromosomal deletions (7q11, 2q12 and 17q24) were examined with oligo array platform and deletion segments of 1.6 (A) and 1.3 (B) mb were detected by this platform but 637 kb (C) was not detected with single cell array.

When samples with known lengths (deletion sizes were 1.6 mb, 1.3 mb and 637 kb, respectively) of chromosomal abnormalities (7q11, 2q12 and 17q24) were labeled with higher resolution oligo chips, we found that the segments that the NimbleGen oligo microarray could detect were 1.3 and 1.6 mb. However, the smaller 637 kb segment could not be detected by this array platform. Thus the NimbleGen 630K chip can detect segments as small as 1.3 mb (Figure 2), while BAC 32K array can detect segments of 4 mb (data not shown), indicating that the NimbleGen oligo array platform is more sensitive than the BAC array platform.

### High proportions of human blastocysts demonstrated aneuploidy, partial chromosome deletions and duplications

**Figure 3:**
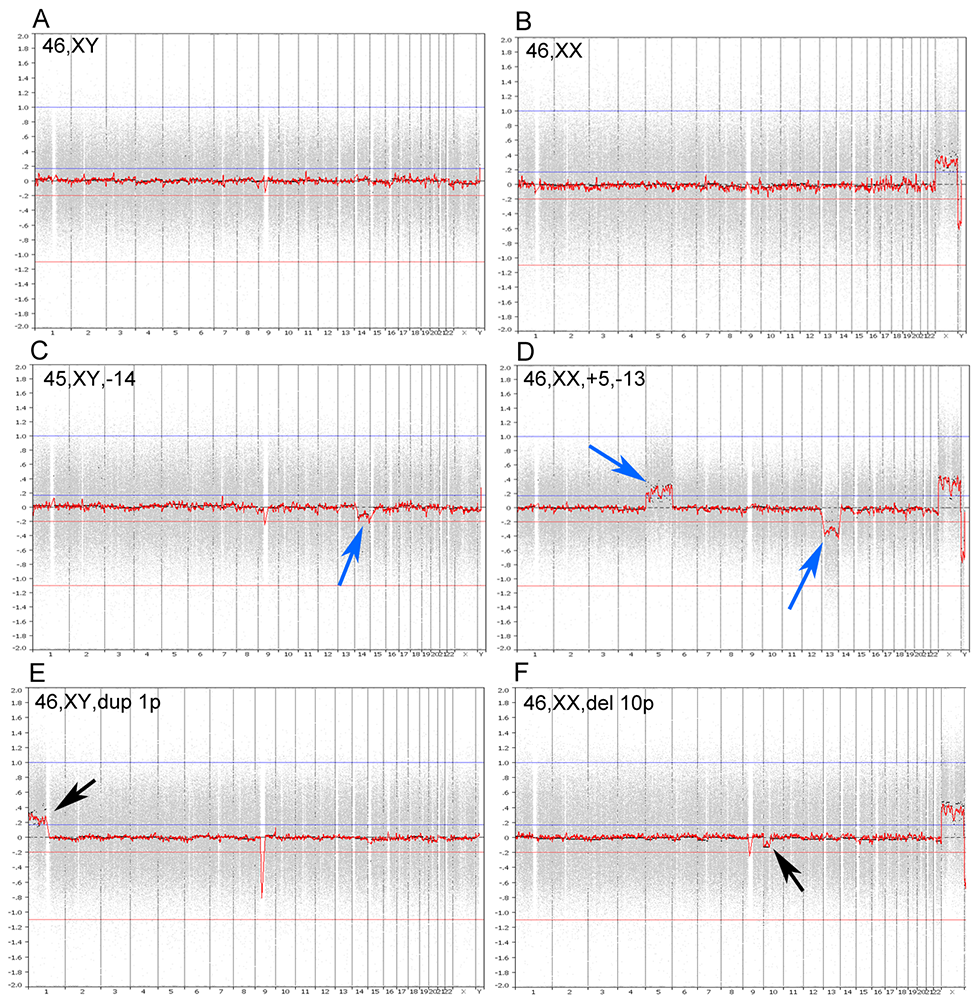
PGS charts of human embryo samples analyzed by NimbleGen microarray platform. (A) normal male (46, XY), (B) normal female (46, XX), (C) single (45, XY, –14) chromosome loss, (D) multiple (46, XX, +5, –13) chromosome errors, (E) euploid cells with partial chromosome duplication (46, XY, dup 1p), and (F) euploid cells with partial chromosome deletion (46, XX, del 10p). Arrows indicate chromosome errors.

Table 1 summarizes the PGS results in the patients studied. In this study, 383 blastocysts were screened for chromosomal abnormalities by microarray and these embryos resulted from 572 fertilized eggs (blastocyst rate of 67.0%) in 72 patients that were either advanced maternal age or had experienced recurrent miscarriage. As shown in Table 1, 98.4% of the samples had DNA signals after microarray. Of these samples, 41.9% were normal euploid blastocysts (Figure 3A and B) and 58.1% were abnormal blastocysts, which included aneuploid blastocysts with a single (Figure 3C) or multiple (Figure 3D) chromosomal errors (gains or losses), and euploid blastocysts with partial chromosome duplications (Figure 3E) or deletions (Figure 3F).

**Table 1:** Microarray analysis of human blastocysts in patients undergoing in vitro fertilization and preimplantation genetic screening.

Observations	Advanced maternal age	Recurrent miscarriage	Total
No. of cycles	51	21	72
Average age	40.2±2.8	33.2±2.2	
No. of zygote (2 pronuclei)	375	197	572
No. (%)* of blastocysts biopsied	246 (65.6)	137 (69.5)	383 (67.0)
No. (%) of samples with DNA signals	242 (98.4)	135 (98.5)	377 (98.4)
No. (%) of normal embryos	87 (36.0)^a^	71 (52.6)^b^	158 (41.9)
No. (%) of abnormal embryos**	155 (64.0)^a^	64 (47.4)^b^	219 (58.1)
No. (%) of patients with embryo(s) for transfer	33 (64.7)^c^	19 (90.5)^d^	52 (72.2)

* Blastocyst rate, out of zygotes (2 pronuclei).

** Abnormal embryos include aneuploidy, chromosome deletions and duplications.

ab P<0.01 within the same row.

cd P<0.05.

**Figure 4:**
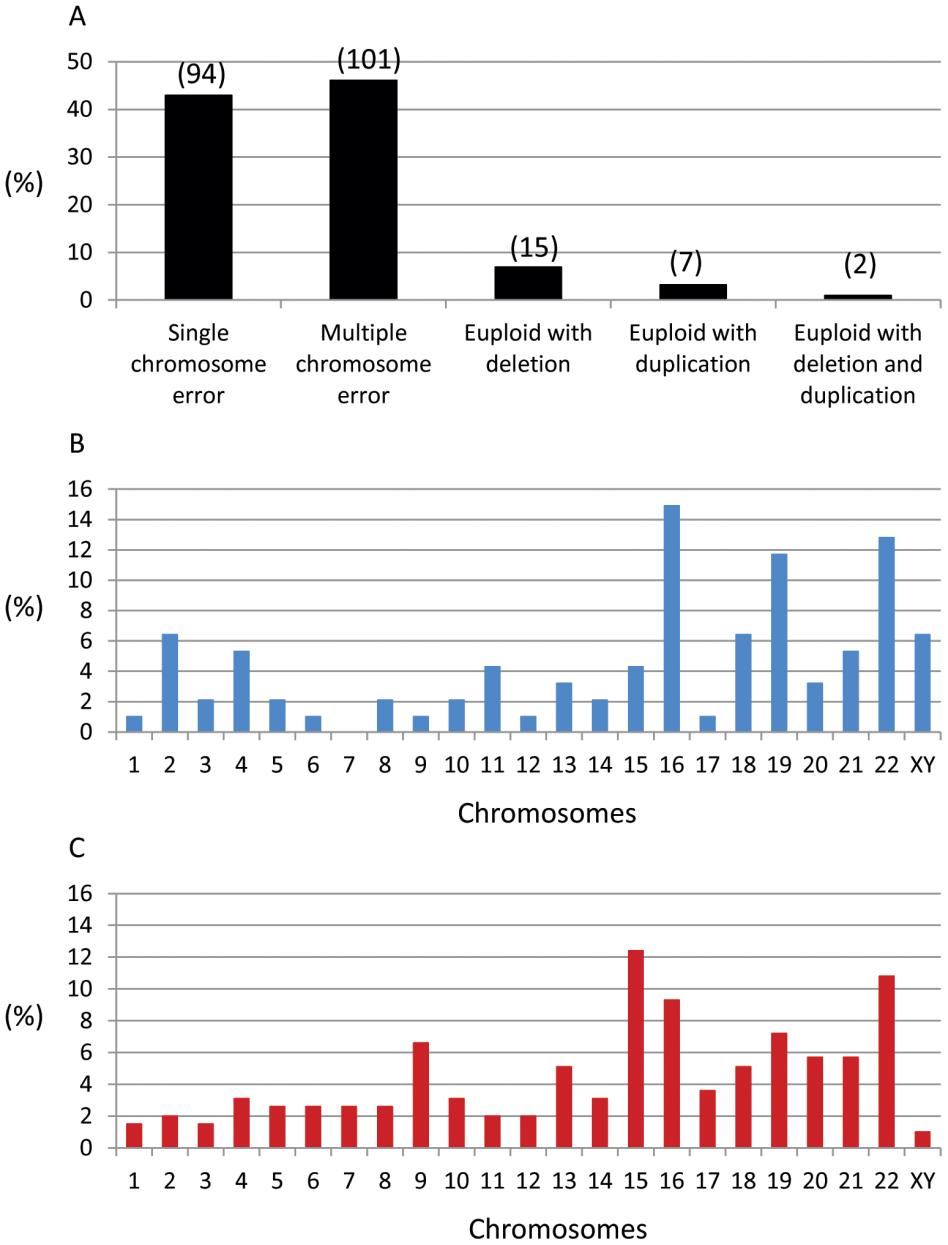
Microarray results of abnormal chromosomes in human blastocysts. (A) abnormal chromosome distribution in the samples examined in the study. Data was based on 219 abnormal samples. Numbers of samples are included in the parenthesis. (B) distribution of a single chromosome error in the abnormal embryos. Data was based on 94 samples. (C) distribution of multiple chromosome errors in the abnormal embryos. Data was based on 197 abnormal chromosomes from 90 samples (11 samples had complex chromosomal abnormalities and were not included in the analysis).

When we compared the chromosomal abnormalities in embryos from patients with advanced maternal age and recurrent miscarriage, we found that patients with advanced maternal age had more abnormal embryos (64.0%) than patients with recurrent miscarriage (47.4%). As shown in Figure 4A, we found that out of 219 abnormal blastocysts, 94 (42.9%) had single chromosome errors, 101 (46.1%) had multiple chromosome errors, 15 (6.9%) were euploid but had partial deletions in some chromosome(s), 7 (3.2%) were euploid but had partial duplications in some chromosome(s) and 2 (0.9%) were euploid but had both partial chromosome duplications and deletions. Partial chromosomal duplications and deletions were also observed in the aneuploid embryos and the data was included in the multiple chromosome error group.

When we further analyzed the detailed chromosomal errors in abnormal embryos, as shown in Figure 4B (single chromosome error) and 4C (multiple chromosome error), chromosome abnormalities occurred in all of the 23 pairs of chromosomes, but it would appear that the errors occurred more frequently in chromosomes 15, 16, 18, 19, 20, 21 and 22.

### Transfer of normal euploid blastocysts resulted in high pregnancy and implantation rates

In the present study, we found that only 64.7% of patients with advanced maternal age had at least one normal transferrable embryo as compared with 90.5% of patients with recurrent miscarriage (Table 1). Twenty patients did not have any euploid embryo for transfer although they had 1–12 blastocysts in each cycle with a total of 69.1% blastocyst formation rate. Out of these 20 patients, most (18) were of advanced maternal ages (Table 2). These results demonstrate that advanced age is a major factor in the formation of aneuploid human embryos.

**Table 2:** Detailed information of human blastocysts analyzed by oligo microarray in the patients without euploid blastocyst.

Case	Age	Patients	No. of fertilized eggs	No. of blastocysts biopsied*	No. of normal blastocysts	No. of abnormal blastocysts
1	40	Advanced maternal age	3	2	0	2
2	42	Advanced maternal age	6	3	0	3
3	34	Recurrent miscarriage	2	2	0	2
4	42	Advanced maternal age	2	1	0	1
5	42	Advanced maternal age	5	1	0	1
6	41	Advanced maternal age	2	2	0	2
7	42	Advanced maternal age	4	3	0	3
8	42	Advanced maternal age	8	7	0	7
9	42	Advanced maternal age	5	3	0	3
10	42	Advanced maternal age	1	1	0	1
11	41	Advanced maternal age	7	2	0	2
12	40	Advanced maternal age	4	4	0	4
13	42	Advanced maternal age	5	4	0	4
14	35	Recurrent miscarriage	12	8	0	8
15	40	Advanced maternal age	5	5	0	5
16	39	Advanced maternal age	3	3	0	3
17	40	Advanced maternal age	10	6	0	6
18	43	Advanced maternal age	7	2	0	2
19	41	Advanced maternal age	12	11	0	11
20	37	Advanced maternal age	7	6	0	6
Total			110	76	0	76

* All blastocysts at day 5 and day 6 were biopsied.

As of now, 34 FETs from 31 patients have been performed, resulting in a 58.8% clinical pregnancy rate and a 54.4% embryo implantation rate. The detailed information, such as patients’ age, number of zygotes, blastocyst formation, chromosomal status and embryo quality, is shown in Table 3. Most patients had transfer(s) with good quality embryos in the present study.

**Table 3:** Detailed analysis of patients who had PGS by microarray and frozen embryo transfer.

Case	Age	No. of fertilized eggs	No. of embryos biopsied*	No. of normal embryos	No. of embryos transferred	Day and quality of embryos**	Clinic pregnancy	No. of embryos implanted
1	41	2	2	1	1	5/G/G	No	0
2.1	39	13	7	2	1	6/F/F	No	0
2.2					1	6/F/F	No	0
3	34	25	20	6	1	6/G/G	Yes	1
4	37	12	5	4	1	5/F/F	Yes	1
5	43	4	3	1	1	6/F/F	No	0
6	41	6	3	1	1	5/G/G	Yes	1
7	37	15	11	5	1	5/G/G	Yes	1
8.1	37	10	5	3	1	5/G/G	No	0
8.2					1	5/G/G	Yes	1
9	33	9	4	2	2	6/G/G, G/F	No	0
10	30	18	10	8	2	5/G/G, G/G	Yes	2
11.1	36	11	10	6	2	5/G/G, G/G	No	0
11.2					2	6/G/G, G/G	Yes	2
12	31	6	3	3	1	5/G/G	Yes	1
13	42	2	2	1	1	5/G/G	Yes	1
14	40	7	4	3	2	6/G/G, G/G	Yes	1
15	34	9	7	3	2	5/G/G,F/F	Yes	1
16	33	1	1	1	1	6/F/F	Yes	1
17	38	20	13	5	1	5/G/G	Yes	1
18	35	9	8	3	1	5/G/G	Yes	1
19	34	9	6	5	2	5/G/G, G/G	Yes	2
20	41	8	3	1	1	5/F/F	No	0
21	40	17	13	6	1	6/G/G	Yes	1
22	37	5	2	1	1	6/F/F	No	0
23	41	13	7	2	2	6/G/G, G/F	No	0
24	48	7	6	4	1	6/G/G	No	0
25	39	3	3	2	2	6/G/G, G/G	Yes	2
26	38	8	6	1	1	6/G/G	Yes	1
27	34	13	4	2	1	6/G/G	Yes	1
28	35	5	4	2	2	6/G/G, G/G	No	0
29	37	13	9	6	1	6/G/G	No	0
30	33	19	18	7	2	5/G/G, G/G	No	0
31	35	7	5	4	2	5/G/G, G/G	Yes	2
Total 34 ET					46		20 (58.8%)	25 (54.4%)

* All blastocysts at day 5 and day 6 were biopsied.

** The number represents blastocyst day, the first letter represents the quality of inner cell mass and the second represents the quality of trophectoderm. G: Good; F: Fair.

Although we obtained a high embryo implantation rate (54.4%), some good embryos still did not implant after transfer. When we investigated the possible reasons for 14 unsuccessful cases, we found that 2 patients had very difficult embryo transfers (transfer took more than 10 min), 3 patients (4 cycles) had fair quality of embryos and others (8) failed due to unknown reasons. When we compared the pregnancy and embryo implantation in women with advanced maternal age and recurrent miscarriage, as shown in Table 4, no differences were observed between the two groups although recurrent miscarriage group had higher rates than advanced maternal age group.

**Table 4:** Clinical outcome of patients who had PGS by microarray and embryo transfer.

Observations	Advanced maternal age	Recurrent miscarriage
No. of cycles	22	12
No. of embryos transfer	27	20
No. (%) of clinical pregnancy *	11 (50)	9 (75.0)
No. (%) of embryos implanted **	13 (48.2)	12 (60.0)

* Clinical pregnancy rate, out of transfer cycles.

** Embryo implantation rate, out of the embryos transferred.

### Accurate aneuploidy assessment with oligo microarray

**Figure 5:**
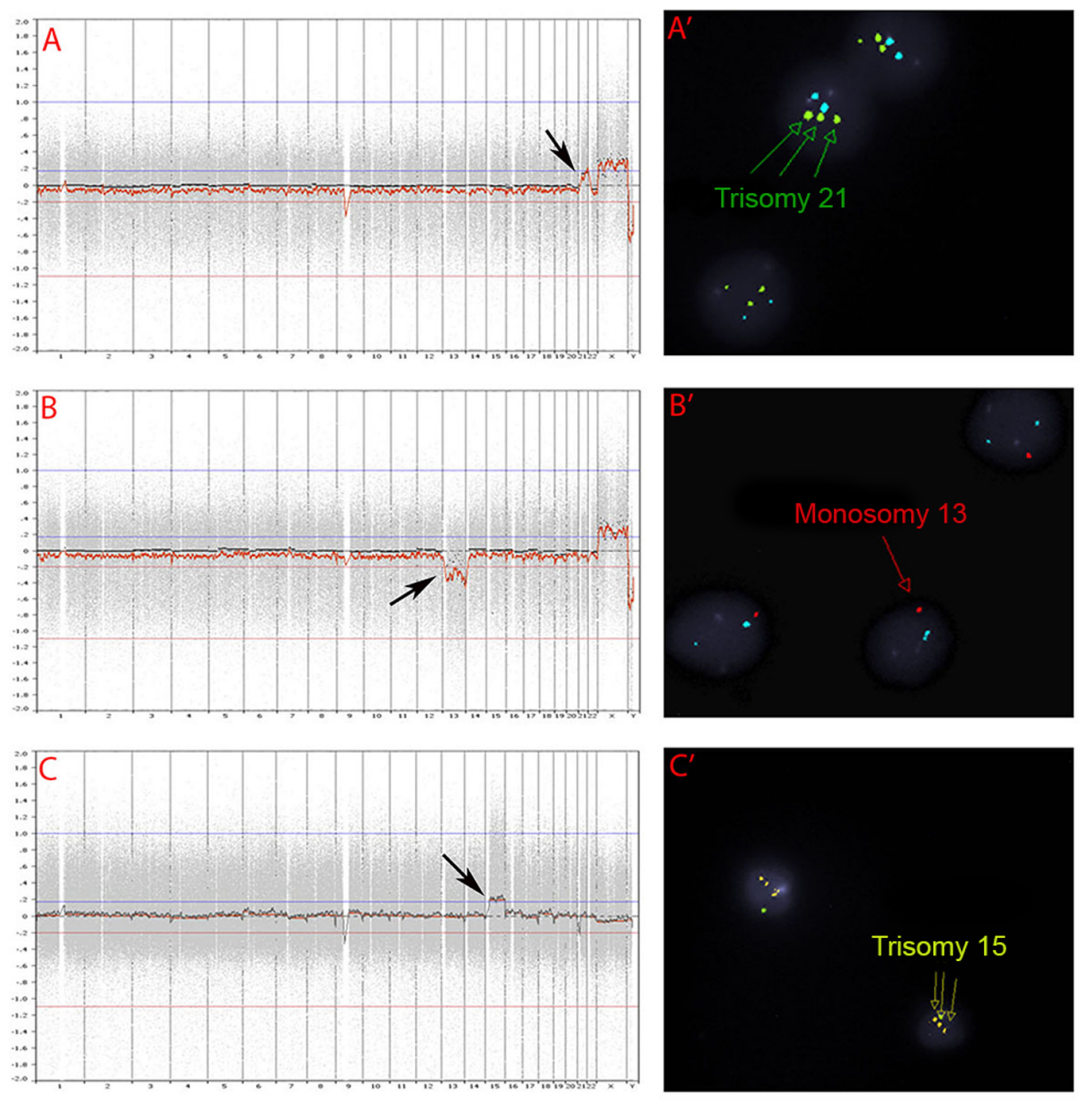
PGS charts and FISH images in aneuploid blastocysts. (A and A’) PGS chart and FISH image of an embryo with 46, XX, +21; Three green staining dots in A’ indicate the presence of three chromosome 21 in the sample and the arrow in A indicates one extra chromosome 21. (B and B’) PGS chart and FISH image of an embryo with 45, XX, –13; One red staining dot in B’ indicates the presence of only one chromosome 13 in the sample and the arrow in B indicates that one chromosome 13 is missing. (C and C’) PGS chart and FISH image of an embryo with 47, XY, +15. Three yellow staining dots in C’ indicate the presence of three chromosome 15 in the sample and the arrow in C indicates one extra chromosome 15.

When 10 aneuploid blastocysts were further analyzed by FISH for the known abnormal chromosomes, all samples had matching results between microarray and FISH (Figure 5).

## Discussion

In the present study, we used a NimbleGen microarray platform that has 630K probes to perform human PGS in patients undergoing IVF. Our results, for the first time, indicate that this NimbleGen oligo platform can be used for PGS in human embryos. The advantages of NimbleGen oligo microarray are its ultra-high density and long oligo probes that enable the highest resolution and most comprehensive array CGH platform for whole genome analysis [20], [22], [23], [26], [28]. Previously, it was found that NimbleGen oligo microarray platform had the best performance after evaluation of the dosage sensitivity, precision, specificity, sensitivity and CNV border definition when comparing different array platforms including BlueGnome BAC platform [20]–[23]. In the present study, we found that this oligo microarray platform can detect segments as small as 1.3 mb, which is more sensitive than the BAC array platform.

DNA microarray is an accurate and reproducible technology for chromosome analysis [12], [16], [18], [31], [32] although different microarray platforms have different features [12], [20]–[23], [27]. In human IVF-PGS services, it has been found that this technology is much more accurate than traditional FISH technology [18], [33], [34], in which the numbers of chromosomes to be analyzed are limited to about five to twelve [5], [35], [36]. For FISH, not only are the numbers of chromosome probes limited, but the probes to cover genomic regions are limited as well. Thus some samples may not be analyzed correctly. Both of these limitations are thought to be significant reasons that previous studies have not shown improved clinical outcomes following transfer of embryos analyzed by FISH [8], [37], [38]. However, with DNA microarray, approximately a few thousands or even a few millions of genomic regions in all chromosomes are able to be measured, thus the error(s) in diagnosis is significantly reduced [12], [16], [18], [31], [32].

In the present clinical investigation, we found that many chromosome abnormalities, such as gains and/or losses of chromosomes, partial chromosome deletions and duplications can be detected by the oligo microarray platform, which revealed that 58.1% of human blastocysts produced by IVF were abnormal. We also found that 24 euploid blastocysts had chromosome deletions (15), duplications (7) or both (2). These results indicate that the oligo microarray platform used in the present study is able to detect not only aneuploidy, but also minor chromosomal abnormalities in the euploid embryos. Although we do not know if transfer of these embryos with minor chromosomal abnormalities would result in birth defects or later disease, it is known that some genetic diseases are indeed caused by partial chromosome duplications or deletions, such as Charcot-Marie-Tooth disease (17p), Canavan disease (17p), Wolf-Hirschhorm Syndrome (4p) and Jackson syndrome (11q) [39].

It has been found that CNVs contribute to inherited genetic disease and to confer resistance to infection, but the full extent of CNVs in the human population and the role of CNVs in some complex diseases, such as diabetes, cardiovascular disease and psychiatric disorders, are not yet understood. It may be possible to use more recent advances in microarray technology to develop more powerful diagnostic tools to study the relationship between minor chromosomal abnormalities in each chromosome and diseases.

Although aneuploidy is the major cause of embryo implantation failure and birth defects [1], some birth defects may be caused by CNVs in the chromosomes [39]. Some array platforms, such as BAC platform, may not be able to detect small CNVs due to fewer probes being used. Theoretically, the more probes are used in an array platform, the more abnormalities can be detected, which makes the NimbleGen platform more powerful. However, the clinical significance of most of these small deletions and duplications is still unknown. Further studies remain necessary to address these questions.

High pregnancy and implantation rates have been reported in humans after transfer of frozen/thawed [13], [14] or fresh [11] blastocysts screened by microarray in women with advanced maternal age [13], [14] or younger patients undergoing IVF [11]. Live births have also been reported in humans after PGS of structural chromosome abnormalities [40], [41] and reciprocal and Robertsonian translocations [42] in which DNA microarray was used. With the widespread clinical applications of DNA microarray in human PGS, more genetic errors in preimplantation embryos would be identified before the embryo transfer, thus the birth defect and/or genetic abnormalities caused by small errors in the chromosomes could be reduced significantly in the future.

In the present study, our results also indicate that PGS by microarray is especially beneficial to patients with advanced maternal ages and recurrent miscarriages as aneuploidy is the major cause of unsuccessful embryo implantation in these patients [1], [2], [6], [7], [9], [42]. In the present study, the aneuploidy rate was very high in patients with advance maternal age. About 35.3% of patients did not have normal embryos for transfer. Like our study, others have shown that high pregnancy and implantation rates, similar to that of younger patients, can be obtained if the patients had normal euploid embryo transfer [11], [13], [14].

Recently, it has been reported that high proportions of aneuploidy were also present in younger IVF patients [10], [11]. Yang et al. [11] obtained higher clinical pregnancy rate in younger women when microarray was used to screen aneuploid embryos than in the patients without using PGS for aneuploidy screening. There is little doubt that aneuploidy is also one of the major reasons for implantation failures in younger patients undergoing IVF as embryo implantation rates are still low (less than 50%). Hence, microarray aneuploidy screening could potentially be beneficial to all patients undergoing IVF. In the present study, we found that a high aneuploidy rate (47.4%) was also present in young patients with a history of previous miscarriage. Transfer of screened blastocysts in these patients also produced very high pregnancy and implantation rates, suggesting that aneuploidy is a significant cause of failed embryo implantation in this group of patients.

The reasons for increased embryonic aneuploid formation in women with advanced maternal age are not fully understood. Many factors contribute to aging related oocyte defects. The general aging process is the major cause [1], [2], [9], [43] but some other external conditions may also contribute to the chromosome instability within oocytes, such as dietary or genetic strategies [44], pollution, metabolic or hormonal problems [45], or medications [45], [46], which in turn cause aneuploidy formation. Procedures for ovarian hyperstimulation and IVF/embryo manipulation may be additional causes for embryonic aneuploid formation in humans [47].

In the present study, when we analyzed the possible reasons for failed embryo implantation in the patients who had normal euploid blastocyst transfer, we found that some embryo implantation failures may have been caused by embryo transfer difficulties and/or fair quality of embryos. However, some other unknown reasons may exist for implantation failure. It would be possible that embryo development beyond blastocyst stage was arrested or uterine-embryo communication was affected due to embryo quality and/or the uterine environment. Further studies are necessary to investigate these reasons so that embryo implantation can be further increased after these problems are solved.

Blastocyst vitrification has become one of the most valuable and important laboratory technologies. When PGS with microarray is performed, especially when microarray is not performed on site, cryopreservation of blastocysts is required for later FET. Previous studies [13], [14] and our current study showed high blastocyst survival rates after vitrification and warming (close to 100%), which contributed to high pregnancy and implantation rates observed in these studies. It is possible that the combination of multiple cell biopsy from blastocysts, all-chromosome microarray, blastocyst vitrification and FET may become a common procedure for IVF-PGS in the future.

## Conclusions

The present results indicate that high proportions of aneuploidy are present in human blastocysts produced by IVF in patients with advanced maternal age and/or recurrent miscarriage. The aneuploidy rate is especially high in the women with advanced maternal age. Oligo DNA microarray can be used to detect most of these chromosome errors, such as aneuploidy, partial chromosome deletions and/or duplications from cells biopsied from blastocysts. Transfer of microarray screened blastocysts can significantly increase clinical pregnancy and embryo implantation rates. After considering the accuracy and the number of genomic probes in the microarray platforms, we expect that oligo microarray platforms with more probes will become one of the most commonly used array platforms in human PGS in the future and its clinical applications will benefit more patients undergoing IVF.
